# Adaptive Multi-Order Penalty and Dual-Driven Weighting: aisPLS Algorithm for Raman Baseline Correction with Weak Peak Preservation

**DOI:** 10.3390/molecules31081243

**Published:** 2026-04-09

**Authors:** Jiawei He, Yonglin Bai, Zishang Jv, Zhen Chen, Bo Wang

**Affiliations:** 1 Key Laboratory of Ultrafast Photoelectric Diagnostic Technology, Xi’an Institute of Optics and Precision Mechanics, Chinese Academy of Sciences, Xi’an 710119, China; hejiawei23@mails.ucas.ac.cn (J.H.); baiyonglin@opt.ac.cn (Y.B.); jzs@opt.ac.cn (Z.J.); 2 State Key Laboratory of Ultrafast Optical Science and Technology, Xi’an Institute of Optics and Precision Mechanics, Chinese Academy of Sciences, Xi’an 710119, China; 3 University of Chinese Academy of Sciences, Beijing 100091, China

**Keywords:** Raman spectra, baseline correction, penalized least squares

## Abstract

Baseline correction of Raman spectra is a critical step for achieving high-precision quantitative analysis. However, the presence of complex background noise, nonlinear baseline drift, and spectral peak distortion due to peak overlap in real spectral data severely limits the performance of conventional correction methods. To better preserve spectral details, this study proposes an improved penalized least squares method for Raman spectral baseline correction. Compared with common baseline correction approaches, the proposed method optimizes the iterative weight function through precise noise classification, significantly enhancing the algorithm’s flexibility. The traditional single smoothing parameter is extended into a smoothing vector, and a classification strategy consistent with that of the penalty parameter is adopted, enabling synchronous optimization and coordinated adjustment of both during iteration. Furthermore, based on the physical constraints of Raman spectra, the algorithm eliminates non-physical solutions that may arise in traditional iterative processes, ensuring the fidelity of the corrected spectra. Experimental results demonstrate that the proposed method exhibits strong robustness under various noise conditions and significantly improves correction accuracy.

## 1. Introduction

Raman spectroscopy, characterized by its high resolution, high accuracy, and rapid real-time analysis capability, is widely employed for qualitative and quantitative analysis in fields such as materials science, food and pharmaceuticals, medical diagnostics, environmental monitoring, and mineral identification [1,2,3,4,5,6,7,8]. However, Raman data analysis faces multiple challenges, among which, baseline drift is a primary concern. Due to the influences of the environment, instrumentation, and the sample itself, Raman spectra frequently exhibit baseline drift. This phenomenon degrades the signal-to-noise ratio, distorts the true shape of spectral peaks, and leads to inaccuracies in the calculation of characteristic parameters such as peak height and peak area, thereby causing significant interference in subsequent qualitative and quantitative analyses [9,10]. Therefore, prior to in-depth analysis, it is essential to remove this background signal through baseline correction algorithms to extract pure, component-related information from complex spectral data. Effectively eliminating the fluorescent background and enhancing data reliability is a prerequisite for accurate band decomposition and all subsequent chemometric analyses.

A variety of baseline correction algorithms are available, including polynomial fitting [11,12,13,14], segmented fitting [15], moving window smoothing [16], wavelet transform [17,18], morphological methods [19,20,21], penalized least squares [22], and deep learning [23]. In the processing of Raman spectra, different baseline correction algorithms exhibit distinct performance characteristics. The derivative method alters the original spectral shape, which can significantly impact subsequent quantitative accuracy. Polynomial filtering employs low-order polynomials to fit the spectrum, yet its parameters require careful selection. In segmented fitting algorithms, the choice of segment points often demands manual intervention, hindering automated baseline correction. The moving window smoothing method iteratively reduces peak intensities but tends to overestimate the baseline, performing particularly poorly in regions with overlapping peaks. Wavelet transform-based correction primarily removes low-frequency components from the spectrum, and its effectiveness heavily depends on the selection of the decomposition scale and the mother wavelet. Morphological algorithms, rooted in image processing, often produce non-smooth baselines and may lead to loss of peak information. Deep learning approaches require extensive training data and involve complex parameter tuning.

The penalized least squares (PLS) algorithm has become one of the most widely used methods for baseline correction due to its high computational efficiency, absence of requirement for peak detection, minimal requirement for prior knowledge, and strong adaptability. The PLS framework was originally introduced by Whittaker in 1922 [24]. Building on this work, various baseline correction algorithms have been developed by adopting different weighting strategies. Eilers et al. proposed the asymmetric least squares (AsLS) method [25]. He et al. subsequently incorporated a first-order derivative constraint to enhance the asymmetric least squares algorithm, resulting in an improved version (IAsLS) [26]. Zhang et al. introduced the adaptive iterative reweighted penalized least squares (airPLS) algorithm [27], which addressed the issue of selecting the asymmetry parameter and made the baseline correction process more automated. Xu et al. proposed the doubly reweighted penalized least squares (drPLS) method [28], which imposes a penalty term to constrain the smoothness term. Baek et al. developed the asymmetric reweighted penalized least squares (arPLS) method [29], which adaptively determines weights using a generalized logistic function to mitigate the influence of noise. Guo et al. fully considered the energy distribution of the signal above and below the fitted baseline, effectively addressing the issue of local over-smoothing [30]. Zhang et al. presented the adaptive smoothing parameter penalized least squares (asPLS) approach [31], which controls the smoothness level through a scaling coefficient.

Although existing PLS-based algorithms have made notable progress, they still suffer from three key limitations. First, their weight update strategies often fail to adequately account for heterogeneous noise distribution, which can lead to the loss of weak broad peaks or overestimation of peak intensities. Second, the use of a single smoothing parameter limits their ability to adapt to varying signal characteristics across different spectral regions, making it difficult to balance baseline smoothness with peak fidelity. Third, the lack of a post-processing mechanism grounded in the physical nature of Raman spectra may introduce non-physical negative signals. To address these limitations, this study proposes the aisPLS algorithm, which incorporates an optimized weight update strategy, an adaptive iterative smoothing vector, and a physically constrained post-processing procedure. These enhancements significantly improve the accuracy and robustness of Raman spectral baseline correction. Comprehensive validation using both simulated and experimentally measured spectra demonstrates that the proposed algorithm achieves excellent performance across varying noise levels and concentration conditions, thereby providing reliable technical support for high-precision quantitative Raman spectroscopy.

## 2. Baseline Correction Results and Analysis of the Simulation Spectrum

In order to evaluate the baseline correction performance of the improved algorithm, this study first conducted validation using simulated Raman spectra. Given that the true baseline of actual Raman spectra is difficult to obtain accurately, making it challenging to reliably assess the correction results, simulated spectra were employed to verify the performance of the proposed method. By constructing simulated data that mathematically model the baseline, noise, and pure spectral signals, it becomes possible to accurately calculate the error between the corrected baseline and the true baseline, thereby providing a reliable basis for comparing the performance of different algorithms.

### 2.1. Simulation of the Raman Spectrum

The simulated spectral data is modeled as three components: the pure spectrum (pr), the baseline (bl), and the noise (ns). The pure spectral component is simulated using Gaussian peaks, incorporating various types of peaks such as overlapping peaks of different shapes, sharp peaks, and weak broad peaks embedded within the noise signal to further evaluate the stability of the algorithm. The baseline component consists of background noise following a Gaussian distribution, along with trigonometric functions, linear functions, exponential functions, and others. Additionally, random noise with varying signal-to-noise ratios is introduced via stochastic functions, enabling a comparison of the baseline correction algorithm’s performance across different SNR conditions.

The Gaussian function is expressed as follows:

(1) $$g\left(x\right)=He^{-{\left(\frac{x-\mu }{\sigma }\right)}^{2}},$$

The Lorentzian function is expressed as follows:

(2) $$l\left(x\right)=H\frac{p_{1}}{{\left(x-\mu \right)}^{2}+p_{2}},$$

where the pure spectrum pr is calculated as a composite of Gaussian and Lorentzian peak functions. The simulated spectrum covers a wavenumber range from 0 cm^−1^ to 1600 cm^−1^. The simulated data is generated according to Equation (3):

(3) $$\begin{array}{c} \;rs\left(x\right)=20e^{-\frac{{\left(x-100\right)}^{2}}{80}}+90e^{-\frac{{\left(x-500\right)}^{2}}{30}}+20e^{-\frac{{\left(x-400\right)}^{2}}{60}}+10e^{-\frac{{\left(x-1100\right)}^{2}}{70}}\\ +10e^{-\frac{{\left(x-1300\right)}^{2}}{70}}+1200\frac{30}{{\left(x-200\right)}^{2}+30^{2}}+300\frac{20}{{\left(x-250\right)}^{2}+20^{2}}\\ +1000\frac{15}{{\left(x-550\right)}^{2}+15^{2}}+1300\frac{20}{{\left(x-600\right)}^{2}+20^{2}}+600\frac{15}{{\left(x-800\right)}^{2}+15^{2}}\\ +500\frac{15}{{\left(x-1000\right)}^{2}+15^{2}}+300\frac{15}{{\left(x-1200\right)}^{2}+15^{2}}\\ +400\frac{15}{{\left(x-1250\right)}^{2}+15^{2}}+200\frac{15}{{\left(x-1450\right)}^{2}+30^{2}}. \end{array}$$

The baseline component is constructed by combining three types of functions: Linear, sinusoidal, and Gaussian, as shown in Equation (4):

(4) $$\{\begin{array}{c} {bl}_{1}\left(x\right)=30+0.0005x,\\ {bl}_{2}\left(x\right)=10\sin\frac{\pi x}{2000},\\ {bl}_{3}\left(x\right)=20\exp(-\frac{{\left(x-700\right)}^{2}}{600^{2}}),\\ bl\left(x\right)={bl}_{1}\left(x\right)+{bl}_{2}\left(x\right)+{bl}_{3}\left(x\right), \end{array}$$

In experimentally measured Raman signals, noise is often present. To simulate the noise encountered in practical spectral data, random functions are employed to introduce noise at signal-to-noise ratios of 20 dB, 40 dB, and 60 dB. The signal-to-noise ratio is defined as follows:

(5) $$SNR=10\mathrm{lg}\left(E_{s}/E_{n}\right).$$

**Figure 1:**
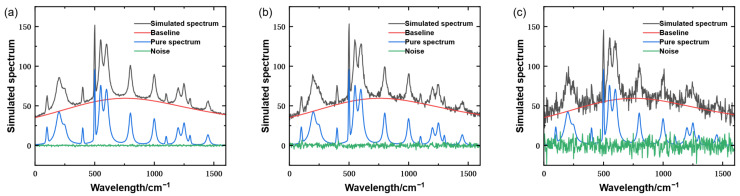
Simulated spectrum under different signal-to-noise ratio conditions. (**a**) Simulated spectrum signal with SNR = 60. (**b**) Simulated spectrum signal with SNR = 40. (**c**) Simulated spectrum signal with SNR = 20.

The resulting simulated spectrum is shown in Figure 1.

### 2.2. Comparison of Different Baseline Correction Algorithms

Table 1 lists the optimal smoothing parameter λ obtained through cross-validation. This procedure ensures that the baseline can effectively suppress background and noise while preserving valid spectral features to the greatest extent, thereby providing a reliable data foundation for subsequent quantitative analysis. The root mean square error (RMSE) is adopted as the evaluation metric, and its calculation formula is defined as follows:

(6) $$RMSE=\sqrt{\frac{\sum_{i=1}^{n}{\left({\hat{b}}_{l}-{bl}_{i}\right)}^{2}}{n}},$$

As evidenced by the quantitative evaluation results in Table 2, the proposed aisPLS algorithm consistently achieves the best baseline estimation performance across various signal-to-noise ratio (SNR) conditions, yielding the lowest root mean square error (RMSE) among all compared methods. This not only demonstrates the stable improvement capability of aisPLS in diverse noise environments, but also highlights its particularly pronounced advantage in low-SNR scenarios—where the algorithm more effectively maintains estimation accuracy despite weak signals and significant noise interference.

**Figure 2:**
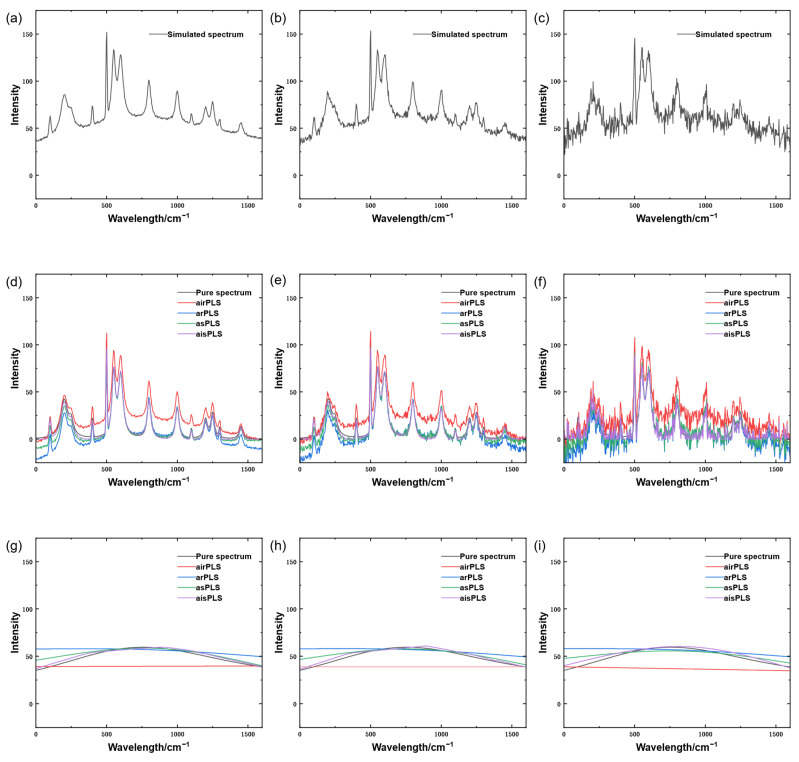
Comparative results of simulated baseline correction. (**a**) Simulated spectrum signal with SNR = 60. (**b**) Simulated spectrum signal with SNR = 40. (**c**) Simulated spectrum signal with SNR = 20. (**d**) Baseline correction results of different algorithms for the simulated spectrum signal with SNR = 60. (**e**) Baseline correction results of different algorithms for the simulated spectrum signal with SNR = 40. (**f**) Baseline correction results of different algorithms for the simulated spectrum signal with SNR = 20. (**g**) Estimated baselines from different algorithms for the simulated spectrum signal with SNR = 60. (**h**) Estimated baselines from different algorithms for the simulated spectrum signal with SNR = 40. (**i**) Estimated baselines from different algorithms for the simulated spectrum signal with SNR = 20.

aisPLS exhibits superior precision in signal decomposition, which is supported by the comparative visualization of baseline correction effects from the four algorithms in Figure 2. The algorithm more clearly separates the baseline component from the effective signal, notably avoiding the common issue of “weak-broad-peak loss” encountered with other methods while effectively suppressing overestimation of strong peaks. In terms of overall fitting quality, aisPLS achieves a better balance between baseline smoothness and spectral fidelity. The estimated baseline curve more closely follows the true background variation, and the corrected spectral profile approximates the ideal state without noticeable distortion or artificial oscillation.

Both quantitative metrics and visual analysis confirm the superior overall performance of aisPLS, particularly its high correction reliability under strong noise interference, which provides robust support for its application in complex real-world scenarios.

## 3. Baseline Correction Results and Analysis for Experimental Raman Spectra

The simulation results in the preceding section have demonstrated the effectiveness of the proposed aisPLS algorithm. To further assess its applicability and reliability in practical detection scenarios, validation experiments were conducted using experimentally measured Raman spectra. Both mineral and organic solution samples were analyzed. The mineral Raman spectra, characterized by weak signals and severe baseline drift, were employed to evaluate the algorithm’s capability in preserving weak spectral features and its accuracy in baseline correction. Meanwhile, the organic solution spectra, despite exhibiting relatively strong signals, still presented noticeable baseline drift, and were thus used to investigate the algorithm’s impact on improving subsequent quantitative analysis.

### 3.1. Raman Spectra of Minerals

The experimental setup employed a prototype Raman spectrometer. The samples tested were polished thin sections of peridotite and pyroxene. The excitation wavelength was 532 nm, with a spectral acquisition range of 200–1200 cm^−1^ and a spectral resolution of 5 cm^−1^. The working distance was set to 30 mm. Each spectrum was acquired with an integration time of 1 s, and 20 acquisitions were averaged to obtain the final result, thereby minimizing random noise interference.

**Figure 3:**
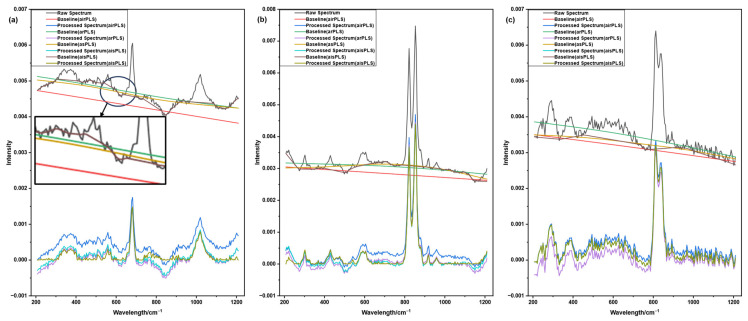
Comparison of raw mineral spectra and the baseline correction results obtained by four algorithms. (**a**) Pyroxene. (**b**) Forsterite. (**c**) Fayalite.

Raman spectra of pyroxene, forsterite, and fayalite were measured experimentally, and the correction results of four baseline correction algorithms were compared. As can be seen from the raw spectra in Figure 3, all three mineral samples exhibit severe baseline drift along with weak signal intensity, with some characteristic peaks being obscured by the baseline drift and noise.

Taking pyroxene in Figure 3a as an example, in the 200–600 cm^−1^ region, the airPLS algorithm exhibits insufficient baseline correction, with the corrected baseline still showing noticeable convex residual drift. In contrast, the arPLS and asPLS algorithms overestimate the baseline, causing the spectrum to be excessively pulled downward. This not only weakens the intensity of the characteristic peak at 682 cm^−1^, but also leads to peak distortion. In the weak signal region of 800–850 cm^−1^, arPLS and asPLS even submerge the effective signals entirely, failing to preserve spectral details. By comparison, the aisPLS algorithm proposed in this study demonstrates superior correction performance: it effectively eliminates baseline drift while perfectly preserving the original morphology of characteristic peaks, without excessive smoothing or peak suppression. Meanwhile, key Raman characteristic peak information at 340 cm^−1^, 373 cm^−1^, and 1015 cm^−1^ is preserved to the greatest extent. These characteristic peaks are essential for the qualitative identification of minerals, and their integrity directly determines the reliability of subsequent analysis.

The baseline correction results for forsterite and fayalite exhibited the same pattern observed in pyroxene. The three comparison algorithms, airPLS, arPLS, and asPLS, all suffered from either over-correction or under-correction, failing to achieve both baseline flatness and preservation of characteristic peak integrity. In contrast, the aisPLS algorithm proposed in this study consistently and effectively eliminated baseline drift while accurately retaining all key characteristic peak information, demonstrating excellent adaptability.

### 3.2. Raman Spectra of Organic Solutions

The fiber optic spectrometer used in the experiment was the DQPro model manufactured by Shanghai RuHai Optoelectronics Technology Co., Ltd. (Shanghai, China), which was equipped with an immersion Raman probe (model RPB4-H). The test liquids were ethanol and acetonitrile mixed at different ratios. Anhydrous ethanol was provided by Tianjin Fuyu Fine Chemical Co., Ltd. (Tianjin, China), while acetonitrile and distilled water were sourced from Xi’an Tianmao Baoding Biotechnology Co., Ltd. (Xi’an, China). All chemicals were used as received without further purification. The excitation wavelength was set at 785 nm, with a spectral acquisition range of 200 cm^−1^–3200 cm^−1^ and a spectral resolution of approximately 5 cm^−1^. To minimize random noise interference, 50 spectral acquisitions were performed for each sample, with an integration time of 1 s per acquisition. The average of these 50 scans was taken as the raw Raman spectrum for each sample. In addition, single-scan spectral data were also collected to simulate real-world rapid detection scenarios.

**Figure 4:**
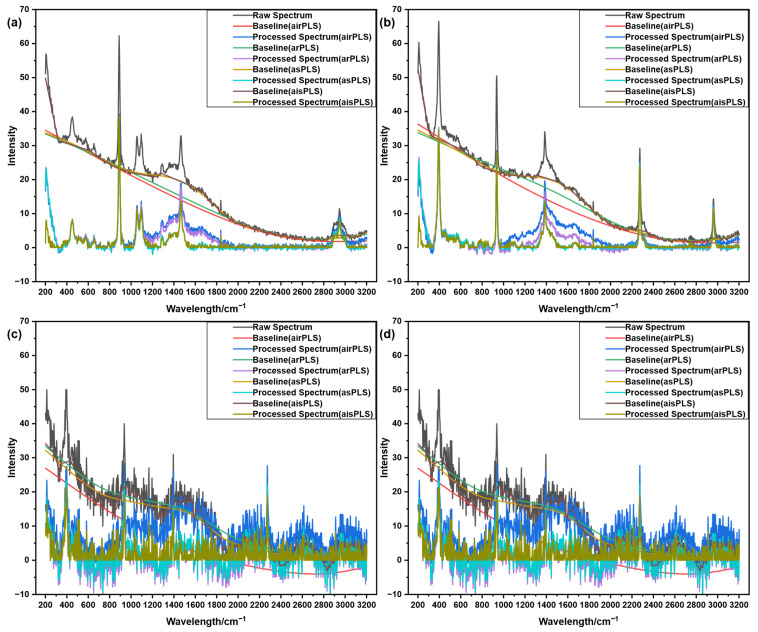
Baseline correction results of different algorithms for Raman spectra of 20% ethanol and acetonitrile. (**a**) Fifty-scan averaged Raman spectra of ethanol. (**b**) Fifty-scan averaged Raman spectra of acetonitrile. (**c**) Single-scan Raman spectra of ethanol. (**d**) Single-scan Raman spectra of acetonitrile.

Figure 4 presents the Raman spectra of 20% ethanol and acetonitrile along with the baseline correction results obtained by different algorithms, including both 50-scan averaged and single-scan measurements, to evaluate the performance of the aisPLS algorithm under varying noise levels. As shown in Figure 4a, the 50-scan averaged ethanol spectrum exhibits significant baseline drift in the 200–250 cm^−1^ region. Among the four algorithms compared, aisPLS demonstrates superior correction performance in this region. However, in the characteristic peak region of 1200–1450 cm^−1^, airPLS and arPLS still suffer from under-correction, which may adversely affect subsequent peak analysis. A similar phenomenon can also be observed in Figure 4b.

Figure 4c,d display the single-scan Raman spectra of ethanol and acetonitrile, which simulate rapid detection scenarios. Due to the short integration time, these spectra exhibit relatively low signal-to-noise ratios. Under such conditions, aisPLS still performs effective baseline correction, and the corrected spectra retain clearer characteristic peak information compared to those processed by other algorithms. This demonstrates the robust performance of the aisPLS algorithm across different signal-to-noise ratio levels, indicating that it exhibits a certain degree of robustness.

**Figure 5:**
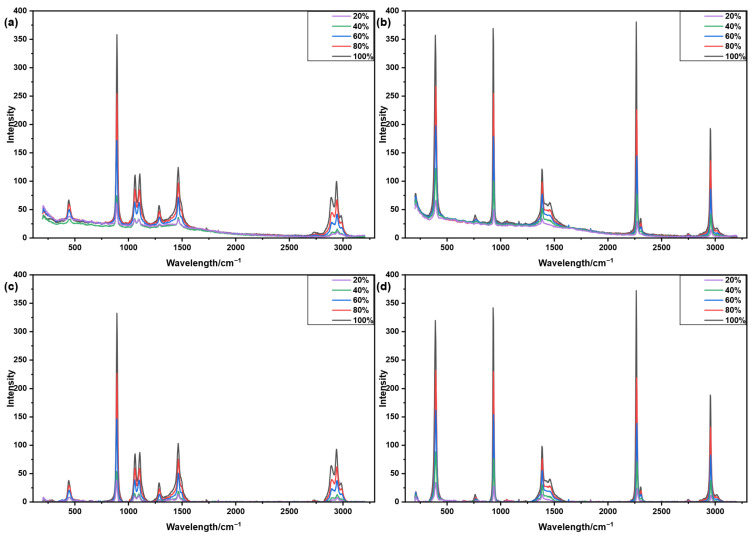
Raman spectra of ethanol and acetonitrile at different concentrations (50-scan averaged) and their baseline correction results using the aisPLS algorithm. (**a**) Raw spectra of ethanol. (**b**) Raw spectra of acetonitrile. (**c**) Corrected spectra of ethanol. (**d**) Corrected spectra of acetonitrile.

**Figure 6:**
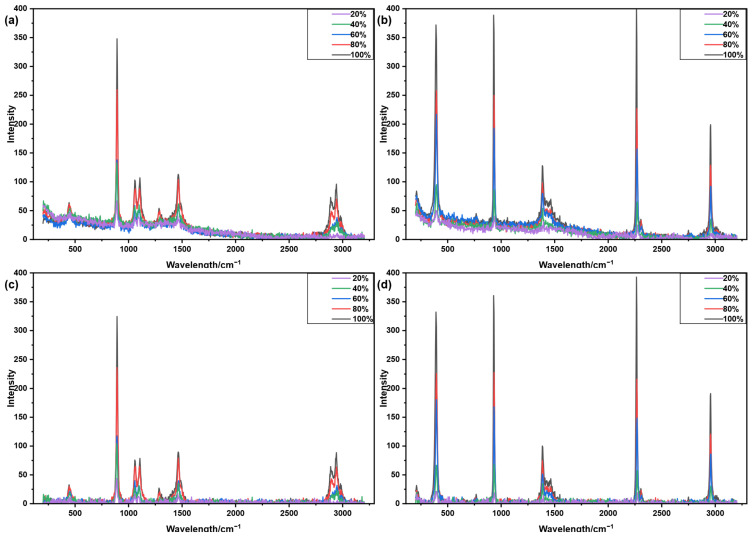
Single-scan Raman spectra of ethanol and acetonitrile at different concentrations and their baseline correction results using the aisPLS algorithm. (**a**) Raw spectra of ethanol. (**b**) Raw spectra of acetonitrile. (**c**) Corrected spectra of ethanol. (**d**) Corrected spectra of acetonitrile.

Figure 5 and Figure 6 illustrate the correction results of the aisPLS algorithm applied to Raman spectra of ethanol and acetonitrile at various concentrations. Figure 5a,b show the raw spectra obtained from 50-scan averaging, while Figure 5c,d present the corresponding corrected spectra. Figure 6a,b display the raw single-scan spectra, with Figure 6c,d showing the spectra after correction. The results demonstrate that aisPLS effectively eliminates baseline drift and restores both the original shape and intensity of characteristic peaks. Across different concentrations and signal-to-noise ratios, aisPLS consistently maintains reliable baseline correction performance for both ethanol and acetonitrile Raman spectra.

Under constant conditions, there exists a linear relationship between Raman scattering intensity and component concentration. Therefore, the improvement in quantitative analysis capability achieved by baseline correction can be evaluated through the linear fitting error (R^2^) of characteristic peak heights. Table 3 compares the R^2^ values of key characteristic peak heights after correction by the four algorithms. The proposed aisPLS method achieves the smallest fitting errors across all characteristic peaks, with R^2^ values of 0.0591 for the C–O bond in ethanol and 0.2194 for the C≡N bond in acetonitrile, significantly outperforming the other methods. This indicates that aisPLS effectively preserves concentration-related quantitative information, facilitating subsequent quantitative analysis.

To further validate the practical application value of the proposed algorithm, we established a partial least squares (PLS) quantitative analysis model based on single-scan Raman spectral data. Compared with the conventional practice of using averaged spectra from multiple scans, modeling with single-measurement data more accurately reflects the algorithm’s performance in real-world rapid detection scenarios. Model performance was comprehensively evaluated using the root mean square error of cross-validation (RMSECV) and the coefficient of determination for prediction (Q^2^). The former reflects the absolute prediction error of the model, while the latter measures the model’s ability to explain variations in sample concentration.

As shown in Table 4, the key performance indicators demonstrate that the model constructed from data corrected by the aisPLS algorithm achieves the lowest RMSECV values (0.0374 for ethanol and 0.0362 for acetonitrile) and the highest Q^2^ values (0.9828 for ethanol and 0.9839 for acetonitrile). It is particularly noteworthy that these results were obtained without spectral averaging—directly processing the raw single-scan signals—further highlighting the ability of the aisPLS algorithm to better preserve the information in the original data. The experimental results clearly indicate that the aisPLS baseline correction algorithm can significantly improve the prediction accuracy and stability of subsequent quantitative analysis models. By effectively extracting high-fidelity spectral features, the algorithm provides solid and reliable technical support for high-precision, rapid quantitative analysis of practical samples using Raman spectroscopy.

## 4. Methodology

### 4.1. The Penalized Least Squares Methods

The PLS algorithm constructs its objective function by jointly constraining the similarity between the signal and the simulated baseline, along with the smoothness of the baseline itself. Assuming the signal sequence is represented as $y={\left[y_{1},y_{2},\ldots ,y_{N}\right]}^{T}$ and the fitted smooth sequence is $z={\left[z_{1},z_{2},\ldots ,z_{N}\right]}^{T}$, the fidelity term F is introduced to quantify the deviation between the fitted sequence and the original signal sequence:

(7) $$F=\sum_{i=1}^{N}{\left(y_{i}-z_{i}\right)}^{2}.$$

The roughness measure R is employed to quantify the smoothness of the fitted sequence. Since second-order differences are often adopted in practical applications, we use first-order difference to simplify the expression of the formula:

(8) $$R=\sum_{i=2}^{N}{\left(z_{i}-z_{i-1}\right)}^{2}={\left\|Dz\right\|}^{2}.$$

Here, Dz=∆z and D is defined as the derivative of the identity matrix and incorporates the smoothing parameter λ. Thus, the cost function Q is formulated as follows:

(9) $$Q=F+\lambda R={\left\|y-z\right\|}^{2}+\lambda {\left\|Dz\right\|}^{2}.$$

During the computational procedure, the baseline estimation problem is transformed into minimizing the cost function, which is typically achieved by setting the partial derivative to zero and solving for the solution:

(10) $$\frac{\partial Q}{\partial z}=-2\left(y-z\right)+2\lambda D^{\prime }Dz=0.$$

The final expression for the fitted baseline z is derived as:

(11) $$z={\left(I+\lambda D^{\prime }D\right)}^{-1}y.$$

where I represents the identity matrix. The standard PLS algorithm does not account for the regional effect of fidelity on the objective function. To address this limitation, the AsLS algorithm introduces a weight vector $W=diag\{\omega_{1},\omega_{2},\ldots \omega_{N}\}$, which aims to assign greater weights to regions with higher signal-to-noise ratios and smaller weights to those with lower signal-to-noise ratios:

(12) $$w_{i}=\{\begin{array}{c} p,\;\;\;\;\;\;\;\;\;\;\;\;\;\;\;\;\;\;y_{i}>z_{i},\\ 1-p,\;\;\;\;\;\;\;\;\;\;\;y_{i}\leq z_{i,} \end{array}$$

The corrected formulation for the fitted baseline **z** is given by:

(13) $$z={\left(W+\lambda D^{\prime }D\right)}^{-1}Wy.$$

By modifying the iterative strategy for updating the weight vector and the termination criteria of the iteration process, various variants of the PLS algorithm have been developed. In the airPLS algorithm, the weight vector is selected according to the following criterion:

(14) $$w_{i}^{t}=\{\begin{array}{c} 0,\;\;\;\;\;\;\;\;\;\;\;\;\;\;\;\;\;\;\;\;\;\;\;\;\;\;\;y_{i}>z_{i}^{t-1},\\ e^{\frac{t\left(y_{i}-z_{i}^{t-1}\right)}{\left|d^{t}\right|}},\;\;\;\;\;\;y_{i}\leq z_{i}^{t-1}, \end{array}$$

where $d^{t}$ denotes a vector composed of elements where $y_{i}-z_{i}^{t-1}<0$. The iteration termination condition is defined as follows:

(15) $$t\geq T\;or\;\left|y-\hat{z}\right|\leq 0.001\times \left|y\right|.$$

In the ArPLS algorithm, the weight vector is updated using the following rule:

(16) $$w_{i}^{t}=\{\begin{array}{c} logistic\left(y_{i}-z_{i},m_{d^{-}},\sigma_{d^{-}}\right)\;,\;\;\;\;\;\;\;y_{i}>z_{i}^{t-1},\\ 1\;,\;\;\;\;\;\;\;\;\;\;\;\;\;\;\;\;\;\;\;\;\;\;\;\;\;\;\;\;\;\;\;\;\;\;\;\;\;\;\;\;\;\;\;\;\;\;\;\;\;y_{i}\leq z_{i}^{t-1}, \end{array}$$

where $d^{-}$ is defined over the range where $y_{i}\leq z_{i}^{t-1}$, with $m_{d^{-}}$ and $\sigma_{d^{-}}$ representing the mean and standard deviation of $d^{-}$, respectively. The logistic function is defined as:

(17) $$logistic\left(d,m,\sigma \right)=\frac{1}{1+e^{2\left(d-\left(-m+2\sigma \right)\right)/\sigma }}.$$

### 4.2. The Proposed Method: aisPLS

The baseline in Raman spectra primarily consists of inherent system noise and background noise. The inherent system noise includes the instrument response function and the dark current of the detector. The system response function typically manifests as the low-frequency component of the spectral curve, which may contain slowly varying tilts or curvatures, while the dark current of the charge-coupled device (CCD) usually presents as positive values. Background noise comprises fluorescence background, scattered light background, and the physical background of the sample, among other sources. The heterogeneity of noise distribution leads to shortcomings in conventional weighting strategies: airPLS directly sets peak regions to zero, which tends to underestimate the baseline and overestimate peak intensities; ArPLS assumes a symmetric noise distribution, resulting in insufficient capability to identify weak and broad peaks, and is prone to over-fitting or under-fitting.

To address the above issues, this study proposes a refined weight update strategy, with two core improvements: first, the application of the 3-sigma rule to eliminate outliers in $d^{-}$, followed by recalculation of the mean m and standard deviation σ, thereby avoiding interference from outliers in weight assignment; second, the introduction of a second-derivative discriminant factor q to achieve refined classification of data points near the baseline, balancing noise suppression with the retention of weak peaks. $D^{2}z$ represents the second derivative of z; q is defined as:

(18) $$q=\left|\frac{D^{2}z}{D^{2}y}\right|.$$

It represents the ratio of the second derivatives of the fitted baseline to the original signal, capturing the abruptness of signal changes at each point. The final weight update rule is formulated as follows:

(19) $$w_{i}^{t}=\{\begin{array}{c} \frac{1}{1+e^{2t\left(d-\left(-m+2\sigma \right)\right)/\sigma }},\;{\;\;\;\;\;\;\;\;\;\;\;\;\;\;\;\;\;\;\;\;\;\;\;\;\;\;\;\;\;\;\;\;\;\;\;\;\;y}_{i}-z_{i}^{t-1}\geq -m+2\sigma ,\\ \frac{1}{1+e^{2t\left(d-\left(-m+2\sigma \right)\right)/q\sigma }},{\;\;\;\;\;\;\;\;\;\;\;\;\;\;\;\;\;\;\;\;\;\;\;\;\;\;0\leq y}_{i}-z_{i}^{t-1}\leq -m+2\sigma ,\\ 1,\;\;\;\;\;\;\;\;\;\;\;\;\;\;\;\;\;\;\;\;\;\;\;\;\;\;\;\;\;\;\;\;\;\;\;\;\;\;\;\;\;\;\;\;\;\;\;\;\;\;\;\;\;\;\;\;\;\;\;\;\;y_{i}<z_{i}^{t-1}, \end{array}$$

The iteration termination condition is similar to (9), ensuring the convergence of the algorithm.

For the smoothing parameter λ, its value typically ranges from 10 to 10^8^, and the specific value must be determined through experimental validation. A larger λ value results in a smoother baseline, whereas a smaller value makes the baseline more susceptible to peak-induced fluctuations. The selection of λ reflects the relationship between the baseline and the signal, directly influencing the accuracy of baseline correction. The final determination of λ requires a combination of theoretical guidance and practical experimental optimization. In the asPLS algorithm, the ratio of the difference between the fitted vector and the spectral signal and its maximum value is used to update λ. However, such an update mechanism may lead to varying λ values even within peak regions, thereby distorting the spectral peak shapes.

To address the above issues, we propose an adaptive iterative smoothing parameter penalized least squares baseline correction algorithm (aisPLS). In aisPLS, a parameter β is introduced as the smoothing parameter adaptation rate, controlling the magnitude of amplification or reduction in each λ_i_ during iterations. The choice of β directly affects the algorithm’s convergence speed, baseline fitting stability, and the preservation of weak peaks. Throughout the iterative process, λ_i_ in peak regions is designed to exhibit an initially slow then accelerating exponential growth, which ensures the integrity of spectral peaks while allowing the baseline in non-peak regions to converge as closely as possible to the true values.

Furthermore, the scalar smoothing parameter λ is extended into a smoothing vector $\lambda =diag\left\{\lambda_{1},\lambda_{2},\ldots \lambda_{N}\right\}$. The initial value is set as $\lambda^{0}=10^{7}$. After initialization, the value of λ at each iteration step t is updated according to the following expression:

(20) $$\lambda_{i}^{t}=\{\begin{array}{c} \lambda_{i-1}^{t}\beta ,\;{\;\;\;\;\;\;\;\;\;\;\;\;\;\;\;\;\;\;\;\;\;\;\;\;\;\;\;\;\;\;\;\;\;\;\;\;\;y}_{i}-z_{i}^{t-1}\geq -m+2\sigma ,\\ \frac{\lambda_{i-1}^{t}}{\beta },{\;\;\;\;\;\;\;\;\;\;\;\;\;\;\;\;\;\;\;\;\;\;\;\;\;\;\;\;\;\;\;\;\;\;\;\;\;\;y}_{i}-z_{i}^{t-1}\leq -m+2\sigma , \end{array}$$

During the baseline correction process using penalized least squares, the corrected Raman spectrum may contain sub-zero signals. Since Raman spectroscopy records the scattering intensity of incident light by the sample, with intensity values representing energy changes in molecular vibrational or rotational modes—which correspond to physically measurable quantities—these values should inherently be non-negative. Based on the slope characteristics of Gaussian and Lorentzian peaks, this study designs a systematic post-processing algorithm. Specifically, the algorithm begins by zeroing all negative-intensity data points. For positive-intensity data points, if the preceding data point was negative, the algorithm evaluates whether the difference between the current positive value and zero is below the standard deviation while further analyzing its variation trend: if three consecutive sampling points fail to exhibit a monotonic increasing or decreasing trend, zeroing is applied. This algorithm ensures spectral fidelity and enhances the accuracy of subsequent Raman spectral analysis. Based on the above discussion, the pseudocode of aisPLS is summarized as Algorithm 1.
**Algorithm 1.** Flow of aisPLS**Input:** spectral data **y**, smoothing parameter $\lambda_{0}$, maximum relative error δ, maximum iteration count T, smoothing parameter adaptation rate r**Output:** Baseline $\hat{b}$, Pure spectral ${\hat{y}}_{p}$(1)Initialization: weight matrix W=diag{1,1,…1}, second-order difference matrix ***D***, Smoothing parameter matrix $\lambda =diag\left\{\lambda_{0},\lambda_{0},\ldots \lambda_{0}\right\},\lambda_{0}=10^{7}$, maximum iteration count T = 200, maximum relative error δ=0.001, smoothing parameter adaptation rate r = 2(2)Weight Update:
(a)calculate the fitted baseline according to Equation (7)(b)compute baseline error d = y − $\hat{b}$, extract elements of d less than 0 to form vector **d**^−^, apply the 3*σ* rule to remove outliers, then recalculate mean m and standard deviation *σ*(c)update the weight matrix **W** according to Equation (13)(3)Simultaneous Smoothing Parameter Update: based on the classification from the weight matrix in Equation (13), update the smoothing matrix λ according to Equation (14)(4)Termination Condition Check: check the termination condition according to Equation (9). If satisfied, proceed to Step (5); otherwise, return to Step (2)(5)Algorithm Termination: output baseline $\hat{b}$, output pure spectrum ${\hat{y}}_{p}=y-\hat{b}$
(a)If $y_{p}<0$, set it to 0(b)If $y_{i-1}<0$, compute $diff1=\left|z_{i}-0\right|$, $diff2=\left|z_{i+1}-z_{i}\right|$, if both diff1<σ,diff2<σ are satisfied, and three consecutive sampling points exhibit no monotonic increasing or decreasing trend, set the corresponding output to 0

## 5. Conclusions

This study proposes an improved baseline correction algorithm for Raman spectroscopy (aisPLS), which introduces three key enhancements to the traditional penalized least squares method: First, an outlier detection and elimination mechanism is incorporated into the weight update process, improving the robustness of the algorithm through statistical analysis. Second, a dual discrimination strategy is designed for data points near the baseline, effectively preserving weak spectral features. Additionally, the smoothing parameter is innovatively extended into an iteratively updated smoothing vector, while a physically constrained post-processing module is integrated to ensure the corrected results are both mathematically sound and physically meaningful.

Simulation experiments demonstrate that the proposed algorithm outperforms existing mainstream methods across various noise levels, with particularly significant improvements in peak recognition accuracy under high signal-to-noise ratio conditions. In practical spectral validation, the algorithm successfully eliminated baseline drift in ethanol and acetonitrile solutions at different concentrations, achieving Q^2^ values above 0.98 for the PLS quantitative models of both substances. The design concept of the algorithm exhibits strong generalizability and can be extended to baseline correction tasks for other spectroscopic techniques such as infrared and fluorescence spectroscopy.

Future work will focus on optimizing the adaptive adjustment mechanism for the smoothing parameter, expanding the algorithm’s applicability to extreme scenarios such as multi-component systems and strong fluorescence backgrounds, and exploring its integration with deep learning methods to develop a more powerful spectral preprocessing framework.

## Figures and Tables

**Table 1:** Selection of the Optimal Smoothing Parameter.

λ	airPLS	arPLS	asPLS	aisPLS
20 dB	$10^{5}$	$10^{6}$	$10^{8}$	$10^{7}$
40 dB	$10^{6}$	$10^{7}$	$10^{8}$	$10^{7}$
60 dB	$10^{5}$	$10^{6}$	$10^{7}$	$10^{7}$

**Table 2:** Root Mean Square Errors of the Four Baseline Correction Methods.

RMSE	airPLS	arPLS	asPLS	aisPLS
60 dB	6.1605	5.604	3.585	2.7194
60 dB	2.177	2.9011	2.1019	1.9116
60 dB	2.6702	2.1912	1.9805	1.8606

**Table 3:** Goodness-of-Fit (R^2^) for Linear Regression of Raman Characteristic Peaks.

Method	airPLS	arPLS	asPLS	aisPLS
C-O (895 cm^−1^)	1.1856	0.162	5.8543	0.0591
C-H (2941 cm^−1^)	15.9998	43.4556	9.4437	2.6674
C-C (929 cm^−1^)	1.0477	1.6342	7.3304	0.3817
C≡N (2264 cm^−1^)	4.2657	1.9407	3.3300	0.2194

**Table 4:** Predictive Performance of PLS Regression Modeling.

Material	Parameter	Method			
		airPLS	arPLS	asPLS	aisPLS
Ethanol	RMSECV	0.0530	0.0446	0.0461	0.0374
	$Q^{2}$	0.9656	0.9757	0.9740	0.9828
Acetonitrile	RMSECV	0.0437	0.0449	0.0442	0.0362
	$Q^{2}$	0.9766	0.9753	0.9760	0.9839

## Data Availability

Data are contained within the article.
